# Pyrexia in the Puerperium

**Published:** 1902-05-10

**Authors:** 


### PYREXIA IN THE PUERPERIUM.

Dr. Ethel Williams[1] draws attention to the considerable degrees of pyrexia which may occur during the puerperium and yet be produced not by uterine but by gastro-intestinal causes. The picture in most minds, when we speak of fever originating in the alimentary tract during the puerperium, is, she says, of a moderate fever of from 100° to 102° Fahr., occurring about the third day with little or no quickening of the pulse or constitutional disturbance—the whole thing vanishing as soon as the bowels are satisfactorily opened; and few people would feel happy in regarding as of purely gastro-intestinal origin cases in which the temperature ran up to 103° to 104° Fahr., with a pulse of 120 to 140. She relates cases, however, which go to show that such may be the case, her object being to suggest that the rôle of the intestinal tube in infections during the puerperium is wider than it is generally supposed to be. It is known, she says, that during the puerperium the resistance of the body against microbic invasion is very markedly diminished, and although some of the cases referred to may be purely sapræmic, it is but natural to believe that this loss of defensive power will be as effective in allowing microbic invasion from the intestinal tube as from its more usual origin the genital tract. Unfortunately, Dr. Ethel Williams cannot find out any definite symptoms marking off these intestinal cases, unless one may rely upon the absence of such as point to the genital organs and the presence of flatulence and pain in the upper quadrants of the abdomen. We may add, however, that the interesting observations which she has made may possibly go far to explain the striking relief which follows the use of saline aperients in some cases which, in default of such distinguishing signs, are often regarded as arising from sepsis in the genital tract; for perhaps so-called puerperal sepsis may be more often intestinal than we imagine or at least may be so far complicated with intestinal sapræmia that by giving relief to that side of the trouble we may enable the patient to face her other difficulties with greater prospect of success.

[1] Lancet, April 19.

